# Presence of the Panton-Valentine Leukocidin Genes in Methicillin-Resistant *Staphylococcus aureus* Is Associated with Severity and Clinical Outcome of Hospital-Acquired Pneumonia in a Single Center Study in China

**DOI:** 10.1371/journal.pone.0156704

**Published:** 2016-06-01

**Authors:** Chuanling Zhang, Liang Guo, Xu Chu, Limeng Shen, Yuanyu Guo, Huali Dong, Jianfeng Mao, Stijn van der Veen

**Affiliations:** 1 Clinical Laboratory, Zhejiang Xiaoshan Hospital, Hangzhou, China; 2 Intensive Care Unit, Zhejiang Xiaoshan Hospital, Hangzhou, China; 3 Department of Microbiology and Parasitology, Collaborative Innovation Center for Diagnosis and Treatment of Infectious Diseases, School of Medicine, Zhejiang University, Hangzhou, China; National Institutes of Health, UNITED STATES

## Abstract

The Panton-Valentine leukocidin (PVL) genes of methicillin-resistant *Staphylococcus aureus* (MRSA) have previously been associated with severe infections. Here, the impact of the PVL genes on severity of disease and clinical outcome of patients with hospital-acquired pneumonia (HAP) or ventilator-associated pneumonia (VAP) due to MRSA was investigated in a single center observational study in a hospital in China. HAP due to MRSA was diagnosed in 100 patients and 13 of the patients were PVL positive, while VAP was diagnosed in 5 patients and 2 were PVL positive. The PVL positive patient group showed a significantly higher Acute Physiology and Chronic Health Evaluation (APACHE) II score (14.3 ±7.8 vs. 10.1 ±4.7, *P* = 0.005) and significantly more patients with CRP levels >80 mg/L (8/15 vs. 12/90, *P* = 0.006) or WBC counts >15x10^9^/L (7/15 vs. 12/90, *P* = 0.006), indicating that the severity of disease is affected by the presence of the PVL genes. The outcome of the study was defined by 30-day mortality. Four (27%) of the PVL positive patients and four (4%) of the PVL negative patients died within 30 days (*P* = 0.01, Fisher exact test). Kaplan-Meier survival curves were generated for the PVL positive and PVL negative patient groups, which differed significantly (*P* = 0.003). Among the patients that died, the mean interval between diagnosis and death was shorter for the PVL positive patients (9.3 ±5.6 vs. 40.8 ±6.6 days, *P* = 0.013). Further analysis within the HAP and VAP patient groups showed that the presence of PVL in MRSA impacted the severity of disease and clinical outcome of HAP, but for VAP the number of patients included in the study was too low. In conclusion, in this single center study in a Chinese hospital the presence of the PVL genes in MRSA impacted the severity of disease and clinical outcome in patients with HAP due to MRSA.

## Introduction

Methicillin-resistant *Staphylococcus aureus* (MRSA) is a major cause of nosocomial infections and is frequently identified in hospital-acquired pneumonia (HAP) and ventilator-associated pneumonia (VAP) [1]. A prevalent virulence determinant of *S*. *aureus* is the Panton-Valentine leukocidin (PVL), which is an exotoxin that creates pores in white blood cells such as neutrophils [2]. Presence of the PVL genes in MRSA isolates from patients with community-acquired pneumonia (CAP) has previously been associated with severe symptoms and poor clinical outcomes [3, 4]. Therefore, treatment with antibiotics that inhibit protein synthesis is currently being considered for CAP patients with PVL positive MRSA [5]. However, the relationship between the presence of PVL genes in MRSA isolates and the severity and clinical outcome of HAP and VAP, which is clinically distinct from CAP, remains elusive and has only been assessed in a few studies. In a retrospective observational study of patients with HAP and VAP in four academic medical centers in the USA, the severity of disease and clinical outcome were not associated with the presence of the PVL genes in MRSA [6]. Similarly, in two multinational clinical trials studying the safety and efficacy of telavancin for the treatment of HAP caused by MRSA, no significant association between the presences of the PVL genes and the clinical outcome of the disease was observed [7]. Both of these studies included patients from multiple centers with a diverse ethnic background. The aim of the present study is to investigate whether the presence of the PVL genes in MRSA has an impact on the severity of disease and clinical outcome in an ethnic homogeneous Chinese patient population with HAP or VAP.

## Materials and Methods

### Study Design

This was an observational study of patients that were diagnosed with HAP or VAP due to MRSA at the Zhejiang Xiaoshan Hospital in Hangzhou (China) from January 2010 to December 2012. HAP and VAP was diagnosed in patients who were in the hospital for at least 48 h and showed both clinical symptoms, including fever (≥ 38.0°C) or hypothermia (≤ 36.0°C), coughs, breathing problems, leukocytosis (≥ 10x10^9^ white blood cells (WBC)/L) or leucopenia (≤ 4x10^9^ WBC/L), oxygen requirements, and purulent sputum or respiratory secretions, and furthermore new or progressive pulmonary infiltrates on chest radiography images. HAP or VAP due to MRSA was identified by at least three positive sputum or tracheal aspirate samples or by a single positive broncho-alveolar lavage sample. At the time of confirmed diagnosis of HAP or VAP due to MRSA, the severity of the disease was assessed using the Acute Physiology and Chronic Health Evaluation (APACHE) II score and by monitoring for clinical parameters that are highly elevated or out of the normal range, such as the C-reactive protein (CRP) levels (>80 mg/L), WBC counts (>15x10^9^/L), urea-to-creatinine ratios (>100), bilirubin levels (>20 μmol/L), and platelet counts (<150x10^9^/L). The clinical outcome of the study was mortality. The study was approved by a local review board at Zhejiang Xiaoshan Hospital. Written informed consent was obtained from all patients, next of kin or caregivers in the case of children to participate in this study. Patient data were collected from the internal Zhejiang Xiaoshan Hospital database after positive identification of MRSA in the patient’s sputum, broncho-alveolar lavage or tracheal aspirate samples. Patient characteristics and underlying diseases included in the analyses were age, gender, diabetes mellitus, cancer, chemotherapy, chronic obstructive pulmonary disease, glucocorticoid use, end stage liver disease, uremia, sepsis, shock, and multiple organ failure. Furthermore, an age adjusted Charlson comorbidity score was calculated for each patient [8].

### Identification of MRSA and the PVL Genes

Patient sputum, broncho-alveolar lavage or tracheal aspirate samples were inoculated on blood agar plates and incubated overnight at 35.5°C in the presence of 5% CO_2_. *S*. *aureus* suggestive colonies were confirmed by coagulase and DNase testing. Methicillin resistance was identified by cefoxitin susceptibility testing. The presence of the PVL genes in the MRSA isolates was identified by PCR and sequencing of *lukSF* using the method described previously [9].

### Molecular epidemiology typing

Multilocus sequence typing (MLST) was performed by PCR and sequencing of seven housekeeping genes using the primers and methods described previously [10]. Sequence types (STs) were generated based on the combination of the specific alleles of the seven housekeeping using the public database (www.mlst.net). In addition, staphylococcal cassette chromosome mec element (SCCmec) types were analyzed by PCR as described previously [11].

### Statistical Analysis

The severity of the disease, the clinical outcome, and the different patient characteristics were compared between patients with HAP or VAP due to PVL positive MRSA and PVL negative MRSA. The distribution of continuous data was analyzed for normality using the Kolmogorov-Smirnov test. Normally distributed data was analyzed using an unpaired parametric two-tailed *t*-test. Data that did not meet the criteria for normal distribution was analyzed with the Mann-Whitney *U* test. Dichotomous data was analyzed using the Fisher exact test. Kaplan-Meier survival curves were generated to compare morbidity and the curves were analyzed using the log-rank test. *P* values of ≤ 0.05 were considered as statistically significant. All statistical analyses were performed with GraphPad Prism software version 6.07.

## Results

### Characteristics of HAP and VAP Patients

In total, 105 patients attending to the Zhejiang Xiaoshan Hospital from January 2010 to December 2012 were diagnosed with HAP (100 patients) or VAP (5 patients) due to MRSA. Investigation of the MRSA strains for the presence of the PVL genes showed that 15 strains (14%) were PVL positive. Of the 15 PVL positive MRSA strains, two were obtained from patients diagnosed with VAP and 13 from patients with HAP. Similarly, three of the 90 PVL negative MRSA strains were obtained from patients with VAP and 87 from patients with HAP. Subsequently, a comparative analysis was performed on the baseline characteristics of the patient groups with HAP or VAP due to PVL positive MRSA and PVL negative MRSA. No significant differences in baseline characteristics or underlying diseases were observed between the two patient groups (Table 1).

**Table 1:** Characteristics of patients with HAP or VAP due to PVL positive and PVL negative MRSA.

Variable	PVL^+^ (n = 15)	PVL^-^ (n = 90)	*P* value
Age (years), mean ±SD	63.7 ±11.3	59.1 ±24.9	0.91^a^
Age (years) ≥ 65, no. (%)	10 (67%)	47 (52%)	0.40^b^
Male sex, no. (%)	11 (73%)	60 (67%)	0.77^b^
Comorbidity score, mean±SD	2.47 ±1.55	2.60 ±1.85	0.79^b^
Diabetes, no. (%)	1 (7%)	8 (9%)	1.00^b^
Tumor, no. (%)	3 (20%)	14 (16%)	0.71^b^
Chemotherapy, no. (%)	0 (0%)	2 (2%)	1.00^b^
COPD, no. (%)	0 (0%)	9 (10%)	0.35^b^
Sepsis, no. (%)	13 (87%)	62 (69%)	0.22^b^
Shock, no (%)	1 (7%)	4 (4%)	0.54^b^
Multiple organ damage, no. (%)	3 (20%)	6 (7%)	0.12^b^
VAP, no (%)	2 (13%)	3 (3%)	0.15^b^
Multilobar positive, no (%)	2 (13%)	26 (29%)	0.34^b^

Abbreviations: PVL: Panton-Valentine leukocidin; SD, standard deviation; COPD, chronic obstructive pulmonary disease.

^a^Mann-Whitney *U* test.

^b^Fisher exact test.

### Severity of Disease

To compare the severity of HAP or VAP due to MRSA that is PVL positive or negative, the APACHE II score was calculated and a range of clinical parameters were evaluated for values that were out of the normal range (Table 2). The APACHE II score was significantly higher in HAP or VAP patients with PVL positive MRSA compared with patients with PVL negative MRSA (*P* = 0.005, unpaired parametric two-tailed *t*-test). However, the APACHE II score has not been validated in patients younger than 16 years of age. This study included nine patients that were younger than 16 years of age, all of them belonging to the PVL negative patient group. After exclusion of these young patients the APACHE II score was still significantly higher for the HAP or VAP patients infected with PVL positive MRSA compared with PVL negative MRSA (14.3 ±7.8 vs. 10.7 ±4.6, *P* = 0.017, Unpaired parametric two-tailed *t*-test). Furthermore, significantly more patients with PVL positive MRSA showed CRP levels >80 mg/L (*P* = 0.006, Fisher exact test) or WBC counts >15x10^9^/L (*P* = 0.006, Fisher exact test) compared with PVL negative patients. These results show that severity of disease in patients with HAP or VAP due to MRSA is associated with the presence of the PVL genes in MRSA.

**Table 2:** Severity of disease in patients with HAP or VAP due to PVL positive and PVL negative MRSA.

Variable	PVL^+^ (n = 15)	PVL^-^ (n = 90)	*P* value
APACHE II score, mean ±SD	14.3 ±7.8	10.1 ±4.7	0.005^a^
CRP >80 mg/L, no (%)	8 (53%)	16 (18%)	0.006^b^
WBC count >15×10^9^/L, no (%)	7 (47%)	12 (13%)	0.006^b^
Platelet count <150×10^9^/L, no (%)	5 (33%)	17 (19%)	0.30^b^
Bilirubin >20 μmol/L, no (%)	5 (33%)	18 (20%)	0.31^b^
Urea-Creatinine ratio >100, no (%)	5 (33%)	28 (31%)	1.0^b^

Abbreviations: PVL: Panton-Valentine leukocidin; SD, standard deviation; APPACHE, Acute, Physiology and Chronic Health Evaluation; CRP, C-reactive protein; WBC, white blood cell.

^a^ Unpaired parametric two-tailed *t*-test.

^b^ Fisher exact test.

### Clinical Outcome

**Fig 1:**
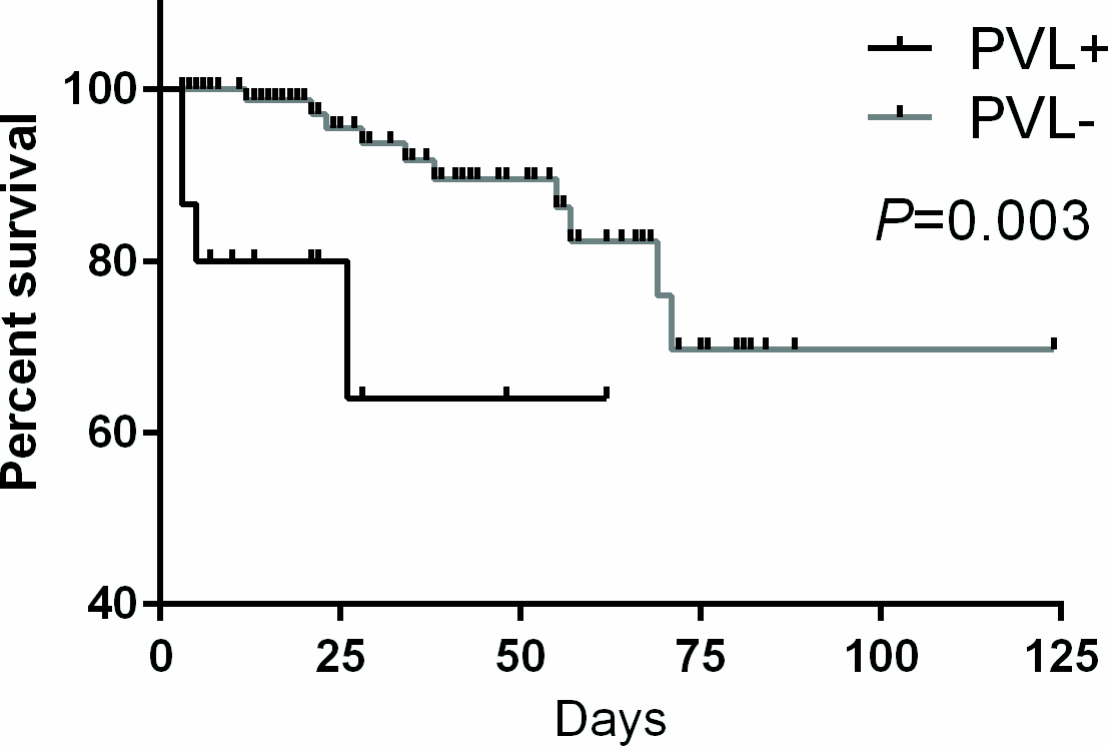
Clinical outcome of patients with HAP or VAP. Kaplan-Meier survival curves for patients with HAP or VAP due to PVL positive and PVL negative MRSA. Curves were compared using the log-rank test and *P* values ≤ 0.05 are considered significantly different.

To determine whether the presence of PVL genes in MRSA affects the clinical outcome of patients with HAP or VAP, 30-day mortality was analyzed. Among the patients infected with PVL positive MRSA, 27% (4/15) died within 30 days, while 4% (4/90) of patients infected with PVL negative MRSA died within 30 days (*P* = 0.01, Fisher exact test). In addition, Kaplan-Meier survival curves that were generated for PVL positive and negative patient groups were significantly different (Fig 1; *P* = 0.003, log-rank test). Among the PVL positive patients who died, the mean interval between diagnosis of HAP or VAP and death was 9.3 ±5.6 days and among the PVL negative patients the mean interval was 40.8 ±6.6 days (*P* = 0.013, Mann-Whitney *U* test). These results show that the prospect of survival is significantly reduced in HAP or VAP patients with PVL positive MRSA compared with patients infected with PVL negative MRSA.

### Molecular Epidemiology Typing of PVL Positive Isolates

To identify whether the association of PVL genes with severity of disease and clinical outcome in patients with HAP or VAP due to MRSA is not the result of high incidence of a specific PVL positive highly virulent clone, MLST and SCCmec typing was performed for all 15 PVL positive MRSA isolates. The 15 PVL positive isolates belonged to six different STs and three different SCCmec types. In addition, for two isolates it was not possible to assign a specific SCCmec type (NT). There appeared to be some bias among our PVL positive isolates, since seven of the isolates were of ST59 SCCmec type III. In addition, three isolates were of ST22 SCCmec type III, two isolates were of ST149 SCCmec type II, one isolate was of ST338 SCCmec type IVa, one isolates was of ST188 SCCmec type NT, and one isolate was of ST88 SCCmec type NT. To investigate the impact of the ST59 SCCmec type III isolates on the association of PVL genes with severity of disease and clinical outcome a thorough comparative analysis of the clinical parameters was performed between the seven PVL positive ST59 SCCmec type III isolates and all 98 other isolates (PVL positive and negative). No significant differences were observed in APACHE II score (12.1 ±6.8 vs. 10.6 ±5.3, *P* = 0.60, Mann-Whitney *U* test), number of patients with CRP levels >80 mg/L (3/7 vs. 21/98, *P* = 0.19, Fisher exact test) or WBC counts >15x10^9^/L (3/7 vs. 16/98, *P* = 0.11, Fisher exact test) or 30-day mortality (1/7 vs. 7/98, *P* = 0.44, Fisher exact test). The seven PVL positive ST59 SCCmec type III isolates were furthermore compared with the eight other PVL positive isolates. Again, no significant differences were observed in APACHE II score (12.1 ±6.8 vs. 16.1 ±8.6, *P* = 0.32, Mann-Whitney *U* test), number of patients with CRP levels >80 mg/L (3/7 vs. 5/8, *P* = 0.62, Fisher exact test) or WBC counts >15x10^9^/L (3/7 vs. 4/8, *P* = 1.0, Fisher exact test) or mortality (1/7 vs. 3/8, *P* = 0.57, Fisher exact test). Finally, reanalysis of the data without the seven PVL positive ST59 SCCmec type III isolates showed that the presence of the PVL genes is still associated with severity of disease and clinical outcome in patients with HAP or VAP due to MRSA. The patient group infected with the eight PVL positive isolates that did not belong to ST59 SCCmec type III showed a significantly higher APACHE II score (16.1 ±8.6 vs. 10.1 ±4.7, *P* = 0.027, Mann-Whitney *U* test), significantly more patients with CRP levels >80 mg/L (5/8 vs. 16/90, *P* = 0.01, Fisher exact test) or WBC counts >15x10^9^/L (4/8 vs. 12/90, *P* = 0.02, Fisher exact test) and a significant higher 30-day mortality (3/8 vs. 4/90, *P* = 0.01, Fisher exact test) than the patient group infected with the PVL negative MRSA isolates. These results indicate that severity of disease and clinical outcome are significantly associated with the presence of PVL genes and not the result an outbreak with a particular highly virulent PVL positive strain.

### Comparative analysis of HAP and VAP patient groups

HAP patients are clinically distinct from VAP patients and therefore these two patients groups were compared for severity of disease and clinical outcome. The VAP patients showed a significant higher APACHE II score compared with HAP patients (16.2 ±6.6 vs. 10.4 ±5.3, *P* = 0.036, Mann-Whitney U test), but no significant differences were observed between the VAP and HAP patient groups in the number of patients with CRP levels >80 mg/L (3/5 vs. 21/100, *P* = 0.078, Fisher exact test) or WBC counts >15x10^9^/L (1/5 vs. 18/100, *P* = 1.0, Fisher exact test). To investigate whether there is a difference in clinical outcome between HAP and VAP patients, 30-day mortality was analyzed. Two of the VAP patients died within 30 days and six of the HAP patients died within this time period. Therefore, 30-day mortality was significantly different between these patient groups (*P* = 0.046, Fisher exact test). These results indicate that the severity of disease and clinical outcome for VAP patients is worse compared with HAP patients. Although the number of VAP patients with PVL positive MRSA did not differ significantly from the number of VAP patients with PVL negative MRSA, data was reanalyzed to investigate the impact of the presence of PVL genes within the HAP and VAP patient groups (Table 3). Within the VAP patient group, no significant differences were observed in any of the clinical parameters or 30-day mortality between patients infected with PVL positive MRSA compared with patients infected with PVL negative MRSA. However, due to the low number of VAP patients included in this study, analyses within the VAP patient group have very low power. More importantly, comparative analyses within the HAP patient group showed that patients infected with PVL positive MRSA still have a higher APACHE II score compared with patients infected with PVL negative MRSA (*P* = 0.011, unpaired parametric two-tailed t-test). Also, the number of patients with CRP levels >80 mg/L (*P* = 0.027, Fisher exact test) or WBC counts >15x10^9^/L (*P* = 0.012, Fisher exact test) was still significantly higher in the PVL positive HAP patient group compared with the PVL negative HAP patient group. Finally, the 30-day mortality was still higher for HAP patients with PVL positive MRSA compared with HAP patients with PVL negative MRSA (*P* = 0.028, Fisher exact test). These results show that the presence of the PVL genes in MRSA impacts the severity of disease and clinical outcome in patients with HAP due to MRSA.

**Table 3:** Severity of disease and clinical outcome in patients with HAP or patients with VAP due to PVL positive and PVL negative MRSA.

Variable	*HAP*			*VAP*		
	PVL^+^ (n = 13)	PVL^-^ (n = 87)	*P* value	PVL^+^ (n = 2)	PVL^-^ (n = 3)	*P* value
APACHE II score, mean ±SD	13.9 ±7.7	9.9 ±4.6	0.011^a^	17.0 ±11.3	15.7 ±4.7	1.0^c^
CRP >80 mg/L, no (%)	6 (46%)	15 (17%)	0.027^b^	2 (100%)	1 (33%)	0.40^b^
WBC count >15×10^9^/L, no (%)	6 (46%)	12 (14%)	0.012^b^	1 (50%)	0 (0%)	0.40^b^
30-day mortality, no (%)	3 (23%)	3 (3%)	0.028^b^	1 (50%)	1 (33%)	1.0^b^

Abbreviations: PVL: Panton-Valentine leukocidin; SD, standard deviation; APPACHE, Acute, Physiology and Chronic Health Evaluation; CRP, C-reactive protein; WBC, white blood cell.

^a^ Unpaired parametric two-tailed *t*-test.

^b^ Fisher exact test.

^c^Mann-Whitney *U* test.

## Discussion

In this study, we investigated the impact of the presence of the PVL genes in MRSA on the severity of disease and clinical outcome in patients with HAP and VAP due to MRSA. A total of 105 MRSA strains from patients with HAP or VAP were analyzed for the presence of the PVL genes and 15 (14%) were found to be PVL positive. This number is higher than the 10% (18/173) PVL positive MRSA strains that were recently identified during a multinational clinical trial for HAP patients [7], but lower than the 27% (29/109) and 22% (55/251) PVL positive MRSA strains identified in patients with HAP and VAP in two recent multistate retrospective observational studies performed in the USA [6, 12].

Our results showed that the presence of PVL genes has an impact on both the severity of the disease and survival of HAP. These results are in contrast to previous studies where no effect of the presence of PVL genes in MRSA on the outcome in patients with HAP or VAP was observed [6, 7]. Both of these studies were multicenter cross-state or multinational studies, while our study was performed at a single hospital, which might explain why we observed a significant effect of the presence of PVL genes in MRSA on the survival of patients with HAP. We furthermore used the APACHE II scores and a range of clinical parameters to evaluate the severity of the disease. The APACHE II score and the number of patients with high CRP levels and WBC counts were significantly higher among the PVL positive group compared with the group of patients with PVL negative MRSA, indicating that the severity of disease is affected by the presence of PVL genes. These parameters are not disease-specific, but they are widely used to measure the severity of disease, tissue inflammation and infection. These results are in contrast with a previous study in which no difference in the APACHE II score was observed due to the presence of PVL genes in MRSA in patients with HAP or VAP due to MRSA [6]. However, besides differences in the study set-up and patient ethnic backgrounds, contrasting results between our study and the studies by Peyrani et al [6] and Sharma-Kuinkel et al [7] might also be explained by the different genetic background of the PVL positive MRSA strains. The PVL positive MRSA strains in the study by Peyrani et al were all of USA300 type and similarly most of the PVL positive isolates in the study by Sharma-Kuinkel et al belonged to clonal complex (CC) 8. USA300 strains are PVL positive ST8 SCC*mec* type IV strains that belong to CC8 [13, 14] and therefore the study by Sharma-Kuinkel et al might be similarly biased towards PVL positive USA300 type strains. Although USA300 PVL positive MRSA strains have previously been associated with more severe infections [15], a recent study suggested that USA300 strains are negatively associated with the severity of clinical courses in CAP [16]. Furthermore, recently it has been indicated that the success of USA300 strains might actually be associated with the acquisition of the *speG* gene [17, 18]. However, a role for *speG* in virulence of MRSA and pathophysiology of pneumonia has not yet been established. In contrast, the PVL positive isolates of our study were not biased towards the USA300 type, which could explain why our study is the first to show positive association between the presence of PVL genes and severity of disease and clinical outcome in HAP and VAP due to MRSA. In our study most PVL positive MRSA strains (67%, 10/15) were of SCC*mec* type III, which is the most common SCC*mec* type found throughout China [19], and only a single PVL positive strain was of SCC*mec* type IV. Furthermore, the 15 PVL positive isolates belonged to six different STs, but there was a bias towards ST59 strains. In the Asia-Pacific region and China, ST59 has become one of the most prevalent MRSA clones [19–23]. Although ST59 strains are most commonly isolated from community associated infections, healthcare associated infections from this ST are also abundant. ST59 MRSA isolates from the Asia-Pacific region and China are also frequently containing the PVL genes [19, 23], although the PVL positive isolates are most commonly of SCC*mec* types IV and V. Since the ST59 PVL positive isolates identified in our study are of SCC*mec* type III, they might be distinct from the common ST59 PVL positive strains in the Asia-Pacific region.

Despite our interesting findings on the association between the presence of PVL in MRSA and severity and clinical outcome of HAP, this study has several limitations. Our findings were obtained in a single center study in a hospital in China, and therefore the results may not reflect the situation in other hospitals or in other countries. Particularly, since the genetic background from the PVL positive MRSA isolates identified in this study differ from those identified in other studies on the role of PVL in MRSA. In addition, this study focused specifically on the role of PVL in MRSA, while methicillin sensitive *S*. *aureus* (MSSA) isolates were disregarded. MSSA isolates also frequently contain the PVL genes and therefore it would be useful to know whether PVL contributes to HAP due to MSSA isolates. Another limitation is that this study included sputum samples for the identification of MRSA. Sputum samples are not fully protected from contamination by oral flora and therefore positive MRSA samples might in some cases have been derived from oral colonization, which could have affected the outcome of this study. Finally, this study has a limited power, since only hundred HAP cases and five VAP cases were included. No power calculation was performed in advance of the study to estimate the number of cases required for statistical analyses and no multivariate analysis was performed. The number of cases included in this study was too limited for a multivariate analysis, particularly for VAP. Therefore, it was not possible to conclude whether PVL is associated with severity and clinical outcome of VAP due to MRSA.

In conclusion, in our single center study in a hospital in China the presence of PVL genes in MRSA impacted the severity of disease and survival of patients with HAP due to MRSA. Although there was a bias in the PVL positive isolates towards ST59, the association between the presence of PVL genes and severity of disease and clinical outcome was not affected by this bias because the association was also observed after omission of the ST59 associated cases.
